# Fluorodeoxyglucose positron emission tomography and chemotherapy-related tumor marker expression in non-small cell lung cancer

**DOI:** 10.1186/1471-2407-13-546

**Published:** 2013-11-15

**Authors:** Xiao-Yi Duan, Wen Wang, Jian-Sheng Wang, Jin Shang, Jun-Gang Gao, You-Min Guo

**Affiliations:** 1 PET-CT Center, the First Affiliated Hospital, Medical School, Xi’an Jiaotong University, No.277 West Yanta road, Xi’an, Shaanxi 710061, People’s Republic of China; 2 Department of Oncology, the First Affiliated Hospital, Medical School, Xi’an Jiaotong University, No.277 West Yanta road, Xi’an, Shaanxi 710061, People’s Republic of China

**Keywords:** Non–small cell lung cancer, Tumor markers, Fluorodeoxyglucose positron emission tomography (FDG–PET)

## Abstract

**Background:**

The chemotherapy resistance of non-small cell lung cancer (NSCLC) remains a clinic challenge and is closely associated with several biomarkers including epidermal growth factor receptor (EGFR) ( Drugs 72(Suppl 1):28–36, 012.), p53 ( Med Sci Monit 11(6):HY11–HY20, 2005.) and excision repair cross complementing gene 1 (ERCC1) ( J Thorac Oncol 8(5):582–586, 2013.). Fluorodeoxyglucose positron emission tomography (FDG–PET) is the best non-invasive surrogate for tumor biology with the maximal standardized uptake values (SUV_max_) being the most important paradigm. However, there are limited data correlating FDG-PET with the chemotherapy resistant tumor markers. The purpose of this study was to determine the correlation of chemotherapy related tumor marker expression with FDG–PET SUV_max_ in NSCLC.

**Methods:**

FDG–PET SUV_max_ was calculated in chemotherapy naïve patients with NSCLC (n = 62) and immunohistochemical analysis was performed for EGFR, p53 or ERCC1 on the intraoperative NSCLC tissues. Each tumor marker was assessed independently by two pathologists using common grading criteria. The SUV_max_ difference based on the histologic characteristics, gender, differentiation, grading and age as well as correlation analysis among these parameters were performed. Multiple stepwise regression analysis was further performed to determine the primary predictor for SUV_max_ and the receiver operating characteristics (ROC) curve analysis was performed to detect the optimized sensitivity and specificity for SUV_max_ in suggesting chemotherapy resistant tumor markers.

**Results:**

The significant tumor type (P = 0.045), differentiation (P = 0.021), p53 (P = 0.000) or ERCC1 (P = 0.033) positivity dependent differences of SUV_max_ values were observed. The tumor differentiation is significantly correlated with SUV_max_ (R = -0.327), tumor size (R = -0.286), grading (R = -0.499), gender (R = 0.286) as well as the expression levels for p53 (R = -0.605) and ERCC1 (R = -0.644). The expression level of p53 is significantly correlated with SUV_max_ (R = 0.508) and grading (R = 0.321). Furthermore, multiple stepwise regression analysis revealed that p53 expression was the primary predictor for SUV_max_. When the cut-off value of SUV_max_ was set at 5.15 in the ROC curve analysis, the sensitivity and specificity of SUV_max_ in suggesting p53 positive NSCLC were 79.5% and 47.8%, respectively.

**Conclusion:**

The current study suggests that SUV_max_ of primary tumor on FDG-PET might be a simple and good non-invasive method for predicting p53-related chemotherapy resistance in NSCLC when we set the cu-off value of SUVmax at 5.15.

## Background

Lung cancer is the most frequently diagnosed cancer and leads to the most cancer mortality worldwide which accounts for almost 1.3 million deaths a year [1]. Nearly 85% of lung cancer cases are represented by non-small cell lung cancer (NSCLC) with the early diagnosis and effective therapy being two main issues [2].

Although significant therapeutic advances have been achieved, poor prognosis and short survival time of patients, as well as the limited value of any sort of conventional therapy are the current dilemma for NSCLC therapy [3]. Platinum-based adjuvant chemotherapy is usually recommended after surgical resection of NSCLC with good performance status and completely resected stage IB-IIIA disease [4]. Such combinational therapy did improve the survival for some patients with early-stage NSCLC [5-7]. However, a large population remains resistant to chemotherapy [8], which has also been confirmed in NSCLC tumor culture study [9]. Increasing evidences advocate the concept that some molecular markers including epidermal growth factor receptor (EGFR) [10], p53 [11] and excision repair cross complementing gene 1 (ERCC1) [12] are associated with chemotherapy resistance in NSCLC. Clarifying the relationship of these molecular markers with noninvasive diagnostic methods is important for the planning of therapeutic strategy.

Fluorodeoxyglucose positron emission tomography (FDG–PET) has become an important non-invasive tool for diagnosing and staging in NSCLC. FDG–PET maximal standardized uptake values (SUV_max_) of primary tumors have been shown to correlate with both stage and nodal disease in NSCLC [13]. Several studies have reported the relationship between the SUV_max_ and the expression levels of some biomarkers, such as Glut 1[14], COX-2[15], Ki-67[16] and vascular endothelial growth factor (VEGF) [17]. Thus we hypothesized that the SUV_max_ of FDG has some close relationship with the chemotherapy resistance associated biomarkers and can serve as a tool to predict some specific chemotherapy résistance for better planning the individualized therapeutic strategy.

The purpose of this study is to examine the relationship between the expressions of chemotherapy resistance related tumor markers and FDG–PET. The SUV_max_ difference based on the histologic characteristics, gender, differentiation, grading and age as well as correlation analysis among these parameters were performed. Multiple stepwise regression analysis was further performed to determine the primary predictor for SUV_max_. Collectively, the current study will offer insight into the relationships between expression of these specific tumor markers and FDG–PET in NSCLC.

## Methods

### Study population

Sixty-two patients with diagnosed NSCLC by biopsy (38/62) or operation (24/62) who were naïve to chemotherapy from the cancer center of our hospital from January 1, 2011 to December 31, 2012 were enrolled in this study. The FDG-PET/CT was performed within one week before biopsy or operation. The histological type was determined according to the World Health Organization (WHO) criteria [18] and the tumor–node–metastasis (TNM) staging system was used according to the criteria in 2011.

Paraffin-embedded primary lung tumor samples were obtained from the pathological department of our hospital. All tissue sections were reviewed for histological type and graded by two pathologists blinded to FDG-PET results. Written informed consent was obtained from each enrolled patient for the study of the excised tissue. This study was conducted with the approval of the institutional ethics committee of the First Affiliated Hospital of Xi’an Jiaotong University.

### ^18^ F-FDG PET/CT

Patients were fasted for 6 hours prior to imaging. FDG-PET images were obtained at 40 min after FDG injection (3.7 MBq /kg) with a PET/CT system (GEMINI 64TF, Philips, Cleveland, USA). Non-contrast CT scan was performed prior to the PET scan with the multidetector spiral CT scanner. PET scan was performed immediately with an acquisition time of 2.0 min/bed position during shallow breathing with the scan field limited from head up to the upper tights. Diagnostic CT scan of chest with respiratory control was performed on the same PET/CT system. Co-registered images were displayed by means of SYNTEGRA software (Philips Medical Systems).

PET/CT images were evaluated by two nuclear physicians in a blinded manner. The SUV_max_ was determined by drawing region of interest (ROI) around the primary tumor on the transaxial slices, and calculated using the following equation: $\frac{\text{tumor}\;\text{activity}\;\text{concentration}}{\text{injected}\;\text{dose}/\text{body}\;\text{weight}}$.

### Immunohistochemical analysis

**Figure 1:**
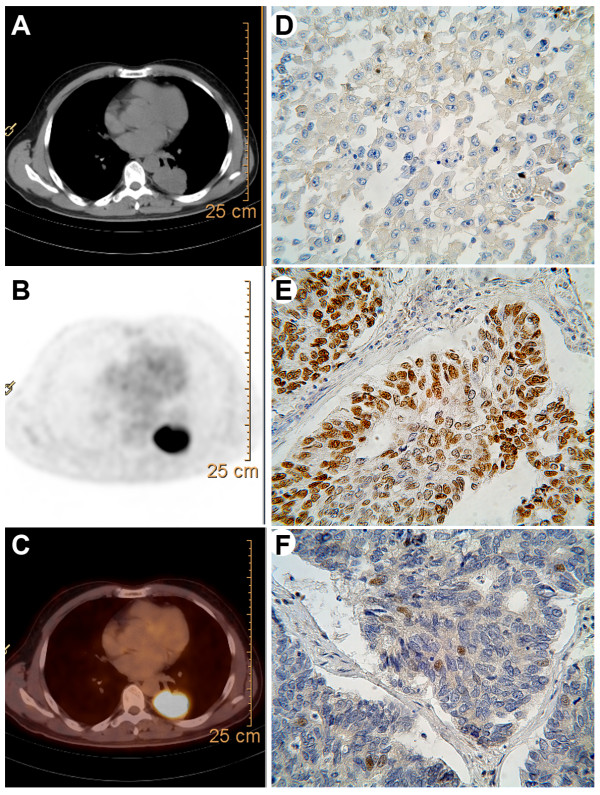
**Representative images of PET-CT and immunohistochemistry.** Transaxial images of **(A)** diagnostic CT, **(B)** FDG-PET and **(C)** fusion of PET and CT images. Immunohistochemical stainings for **(D)** epidermal growth factor receptor (EGFR), **(E)** p53 and **(F)** excision repair cross complementing gene 1(ERCC1). (magnification, ×400).

Immunohistochemical analysis was performed on paraffin-embedded lung cancer tissues. Information of the antibody, dilution and staining pattern were summarized in Table 1. The sections were examined by 2 investigators who had no knowledge of the corresponding clinical pathologic data. For p53 (Figure 1E) and ERCC1 (Figure 1F), nucleus and/or cytoplasm staining was considered positive. EGFR was considered positive when cell membrane and/or cytoplasm staining was observed (Figure 1D). Intensity of staining was scored as the following: 0 (no staining), 1+ (weak staining), 2 + (intermediate staining), 3 + (strong staining). The percentage of positive cells was scored as 0 (0%), 1 (1% to 9%), 2 (10% to 49%), and 3 (50% to 100%) for ERCC1 and p53. For EGFR, it is 0 (0%), 1 (1% to 9%), 2 (10% to 25%), and 3 (>25%). The immunohistochemistry (IHC) score (0 to 9) was defined according to the product intensity and percentage of positive cells. We categorized the patients into four groups according to IHC score (0, 1 to 3, 4 to 6, 7 to 9). The biomarkers expression was judged as positive when the IHC score was greater than or equal to 1 (groups 2, 3 and 4) (Figure 1D, E and F). EGFR, p53 and ERCC1 were positive in 43.5%, 62.9% and 67.7% NSCLCs.

**Table 1:** Antibodies used for immunohistochemical analysis

**Antibody**	**Company**	**Catolog#**	**Clone**	**Dilution**	**Positive staining pattern**
EGFR	Invitrogen	ZM-0083	31G7	1:100	Cytomembrane/Cytoplasm
p53	Invitrogen	ZM-0408	BP53.12	1:50	Nucleus
ERCC1	Invitrogen	ZM-0138	4 F9	1:50	Nucleus/Cytoplasm

EGFR = epidermal growth factor receptor; ERCC1 = excision repair cross complementing gene 1.

### Statistical analysis

Statistical analysis was performed using SPSS software, version 17.0 (SPSS Inc, Chicago, IL). The results were expressed as mean ± standard error mean (SEM). The age, tumor size, p53 positivity and ERCC1 positivity dependent differences were tested using student t-test or one way analysis of the variable (ANOVA) followed by LSD *post hoc* test. Spearman correlation analysis was used to determine the relationship between different parameters. To identify the primary predictor for SUV_max_, multiple stepwise regression analysis was performed. Receiver operating characteristics (ROC) curve analysis was generated that maximized the sensitivity and the specificity and thus the accuracy for assessing a cut off value for SUV_max_ ratio. Differences were considered significant when the P value was less than 0.05.

## Results

### Clinical characteristics

**Figure 2:**
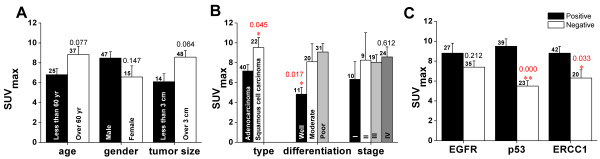
**Group difference of SUV**_**max**_**.** The SUV_max_ differences among the patients with different **(A)** age, gender and tumor size; **(B)** cancer type, differentiation, stage as well as **(C)** expression of 3 biomarkers.** P < 0.01; * P < 0.05; # in (B)^*^P < 0.05 vs well-differentiation group; SUV_max_ = maximal standardized uptake value.

The characteristics of the patients are summarized in Table 2. The patients’ age ranged from 33 to 81 years (median age, 62 years). There were 47 men (median age 65 years) and 15 women (median age 60 years) and there was no difference in ages of these 2 groups (P = 0.095). The median values of the SUV_max_ were 7.2 (range, 1 to 20.8), 7.8 (range, 2.2 to 20.8) and 5.7 (range, 1 to 17.1) in the total, male, and female populations, respectively. Histological type of NSCLC fell in adenocarcinoma (n = 40) and squamous cell carcinoma (n = 22). No significant difference in SUV_max_ of the groups with different age (P = 0.077), gender (P = 0.147) or tumor size (P = 0.064) was observed (Table 2, Figure 2).

**Table 2:** **Characteristics of the patients with SUV**_**max**_

**Factor**	**All patients (n = 62)**	**SUV**_**max**_**(mean ± SEM)**	**P value**
Age			0.077
< 60	25	6.81 ± 0.61	
≥ 60	37	8.83 ± 0.83	
Gender			0.147
Male	47	8.48 ± 0.64	
Female	15	6.57 ± 1.13	
Tumor size (diameter)			0.064
< 3 cm	14	6.10 ± 0.80	
≥ 3 cm	48	8.58 ± 0.67	
Tumor differentiation			0.021
Well	11	4.85 ± 0.69	
Moderate	20	8.10 ± 1.81	
Poor	31	9.09 ± 0.85	
Tumor type			0.045
Adenocarcinoma	22	9.53 ± 1.01	
Squamous cell carcinoma	40	7.19 ± 0.64	
Stage			0.612
I	10	6.35 ± 1.77	
II	9	8.27 ± 2.76	
III	19	8.06 ± 0.77	
IV	24	8.59 ± 1.01	

### The age, tumor size, p53 positivity and ERCC1 positivity dependent differences in SUV_max_

Student t-test and one way ANOVA were performed to determine the parameter based group differences in SUV_max_ (Table 2, Figure 2). In the current study, Student t-test revealed significantly higher SUV_max_ in the patient population with squamous cell carcinoma (P = 0.045), p53 positive (P = 0.000) or ERCC positive cancers (P = 0.033), respectively.

One way ANOVA revealed significant difference in the mean SUV_max_ of NSCLC with different differentiation [F (2,61) = 4.126, P = 0.021]. LSD *post hoc* test revealed that the difference was derived from the significantly higher SUV_max_ from the NSCLC patients with poor (P = 0.017) differentiation. There was no significant difference in the SUV_max_ from poorly and moderately differentiated tumors (P = 1) or moderately and well differentiated tumors (P = 0.132). On the other hand, no difference in the SUV_max_ of patients at different clinical stages [F (3,61) = 0.608, P = 0.612] was observed (Figure 2).

### Correlationship analysis among the parameters

**Figure 3:**
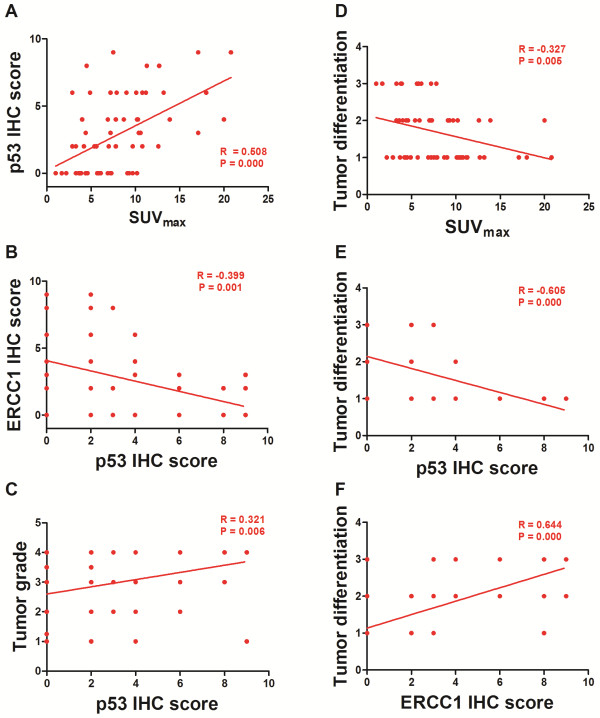
**Correlationship analysis among the parameters.** SUV_max_ was significantly correlated with p53 IHC score (**A**, R = 0.508, P = 0.000) or tumor differentiation (**D**, R = -0.327, P = 0.005). The IHC score of p53 was significantly correlated with that of ERCC1 (**B**, R = -0.399, P = 0.001), tumor differentiation (**E**, R = -0.605, P = 0.000) or clinical stage (**C**, R = 0.321, P = 0.006). Furthermore, tumor differentiation was significantly correlated with ERCC1 IHC score (**F**, R = 0.644, P = 0.000).

Table 3 demonstrated the correlationship analysis among the parameters. SUV_max_ was significantly correlated with p53 IHC score (R = 0.508, P = 0.000, also see Figure 3A) or tumor differentiation (R = -0.327, P = 0.005, also see Figure 3D). Besides SUV_max_, p53 IHC score was significantly correlated with ERCC1 IHC score (R = -0.399, P = 0.001, also see Figure 3B), tumor differentiation (R = -0.605, P = 0.000, also see Figure 3E) or clinical stage (R = 0.321, P = 0.006, also see Figure 3C). Furthermore, tumor differentiation was significantly correlated with other factors including ERCC1 IHC score (R = 0.644, P = 0.000, also see Figure 3F), tumor long axis (R = -0.323, P = 0.006), clinical stages (R = -0.499, P = 0.000) or gender (R = 0.286, P = 0. 013).

**Table 3:** Correlation analysis among different parameters

		***SUV***_***max***_	***p53***	***ERCC1***	***Tumor size***	***Long***	***Differentiation***	***Grading***	***Age***	***Gender***
*Pearson correlation*	*SUV*_*max*_	1.000	.508**	-.067	.174	.206	-.327**	.143	-.118	-.168
	*p53*	.508**	1.000	-.399**	.158	.196	-.605**	.321**	-.106	-.191
	*ERCC1*	-.067	-.399**	1.000	-.181	-.175	.644**	-.241*	.093	.240*
	*tumorsize*	.174	.158	-.181	1.000	.920**	-.286*	-.017	.170	-.112
	*long*	.206	.196	-.175	.920**	1.000	-.323**	-.069	.127	-.077
	*differentiation*	-.327**	-.605**	.644**	-.286*	-.323**	1.000	-.499**	.197	.286*
	*grading*	.143	.321**	-.241*	-.017	-.069	-.499**	1.000	-.048	-.206
	*age*	-.118	-.106	.093	.170	.127	.197	-.048	1.000	-.030
	*gender*	-.168	-.191	.240	-.112	-.077	.286*	-.206	-.030	1.000

*, P < 0.05; **, P < 0.01.

Based on the findings that p53 IHC level was closely related with SUV_max_ and ERCC1 positive tumors demonstrated significantly higher SUV_max_, it is reasonable to hypothesize that SUV_max_ might be usable in predicting the p53 or ERCC1 related chemotherapy resistance. Thus, we performed the multiple stepwise regression analysis to determine which molecule is the primary predictor for SUV_max_.

### IHC score of p53 is the primary predictor for SUV_max_

Employing the multiple stepwise regression model, we input the SUV_max_ as the dependent variable, all the other parameters including age, gender, tumor size, differentiation, clinical stage, IHC score for p53 and ERCC1 as the independent variables. Multiple stepwise regression analysis revealed that the adjusted R^2^ for p53 IHC score is 0.246 and the P value is 0.000 (Table 4, Additional file 1: Figure S1). This statistical finding strongly suggests that p53 IHC score is the primary predictor for SUV_max_. In another word, the SUV_max_ reflects the expression level of p53, thus may offer useful information for the p53 related chemotherapy resistance.

**Table 4:** **Multiple stepwise regression analysis of primary predictor for SUV**_**max**_

**Model**	**R**	**R**^**2**^	**Adjusted R**^**2**^	**SE of the Estimate**	**Change statistics**				
					**R**^**2**^**Change**	**F Change**	**df1**	**df2**	**Sig. F Change**
MSR	.508	.258	.246	3.84094	.258	20.562	1	59	.000

Predictor: p53; Dependent Variable: SUV_max_; MSR: Multiple Stepwise Regression; SUV_max_ = maximal standardized uptake value.

The SUV_max_ greater than 2.5 is often used as a cut-off value for malignancy. However it has been shown that there is a significant number of false positivity (due to inflammatory diseases) and false negativity (due to low-grade malignancies) in the evaluation of primary tumor [19]. A recent study suggested that the cut-off value of SUV_max_ larger than 5 leads to an optimized diagnosing sensitivity and specificity of NSCLC [20]. We thus investigated the sensitivity and specificity at these two cut-off values.

**Figure 4:**
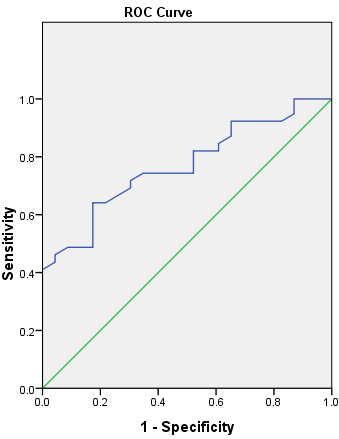
**The receiver operating characteristics (ROC) curve for the optimal cut-off value of SUV**_**max **_**in suggesting p53 positive NSCLC.** Area under the curve: 0.769; 95% CI: 0.654 to 0.884; p = 0.000. A SUV_max_ ratio of 5.15 or lower suggests a NSCLC to be p53 positive with a sensitivity of 79.5% and specificity of 47.8%.

ROC curve analysis revealed that the area under the curve is 0.769 with the 95% confidence interval (CI) ranging from 0.654 to 0.884 (p = 0.000). When the cut-off value of SUV_max_ was set at 2.55, the sensitivity and specificity of suggesting p53 positive NSCLC were 100% and 13%, respectively. However, when we set the cut-off value of SUV_max_ at 5.15, the sensitivity and specificity of suggesting p53 positive NSCLC were 79.5% and 47.8%, respectively (Figure 4).

## Discussion

FDG-PET, one of the current-available non-invasive imaging methods, has long been used to determine the enhanced metabolism in malignant tumor indicated by increased glucose uptake which is represented by an increased SUV_max_. Our study offers further evidence that the SUV_max_ of FDG-PET may be a predicting parameter for some chemotherapy resistant NSCLCs, especially for the p53 or ERCC1 related chemotherapy resistance. Furthermore, SUV_max_ may be the most relevant parameter for p53 related chemotherapy which suggests the future clinical application to design the therapeutic plan.

EGFR is a cell surface receptor found primarily on cells with epithelial origin. EGFR overexpresses in both cell lines and samples of NSCLC, and contributes to the increased tumor proliferation, poor differentiation, higher incidence of metastases to lymph nodes and a worse prognosis [21]. Previous studies have demonstrated that expression status of EGFR can predict treatment response and survival benefit from the addition of cetuximab to first-line chemotherapy in patients with advanced NSCLC [11]. Taylor and colleagues [22] found that there was no correlation between SUV_max_ and EGFR expression in esophageal cancer specimens. Shimizu et al [15] reported that phosphorylated EGFR-positive cases showed higher SUV_max_ than negative cases in lung adenocarcinoma, but without statistical significance. Our finding is quite consistent with theirs in that there is no relationship between EGFR expression and SUV_max_ in NSCLCs. Furthermore, we did not reveal any difference in the SUV_max_ between adenocarcinoma and squamous cell carcinoma. Our study, together with previous one [15], suggests that FDG-PET may not be suitable for determining EGFR-related chemotherapy resistance or evaluating therapeutic effect of anti-EGFR treatment for NSCLCs.

The anti-cancer mechanism for the platinum compounds is to form adducts and covalent cross-links between DNA double strands and thus effectively block DNA replication and transcription. ERCC1 can recognize and remove these adducts and covalent cross-links, thus resistant to platinum agents [12]. A recent meta-analysis indicated that high ERCC1 level was a positive prognostic factor, being associated with shorter survival and lower response to platinum-based chemotherapy in advanced NSCLC patients [23]. Interestingly, we revealed that the SUV_max_ of ERCC1-positive cases were significantly higher than that of ERCC1-negative cases, there was statistical correlation between SUV_max_ and ERCC1 level, but failed to detect robust correlationship when the multiple stepwise regression was performed. It is still inconclusive whether SUV_max_ can be used to determine ERCC1 related chemotherapy resistance based on our current study.

As a tumor suppressor gene, p53 is capable of either arresting the cell cycle or inducing apoptosis. Tumors expressing p53 were less resistant to cisplatin, carboplatin, paclitaxel, and gemcitabine [10], probably due to the transcription of some MDR genes in these tumors [24]. A previous study suggested that there was no association between p53 expression and FDG uptake in 23 resected NSCLCs [25]. This is inconsistent with our current finding that the mean SUV_max_ of p53-positive cases was statistically higher than that of p53-negative cases. Besides, we also offered evidence that p53 expression is the primary predicting factor for the SUV_max_. Our findings lead to the concept that FDG-PET can be used to represent p53 expression status, thus predict the p53-related chemotherapy sensitivity. In the clinic settings, we should set the cut-off value of SUV_max_ at around 5 to get the optimized sensitivity and specificity However, this is a more like bench study even if we used the clinical imaging technique. Using p53 as the biomarker for chemotherapy resistance in NSCLC is risky. Thus, cautions should be taken when using the SUV_max_ of FDG as an alternative or reliable marker for p53, not to mention the prognosis of NSCLC. To really apply the SUV_max_ of FDG in the clinic settings, more bench studies and clinic trials are needed. Further efforts are needed to reveal the underlying reasons for the inconsistency between the findings of ours and others, the study to fill the gap between our experimental findings and future clinical applications should also be considered.

## Conclusions

In conclusion, the expressions of p53 and ERCC1 are associated with the SUV_max_ on FDG-PET in NSCLCs. Of the two markers, p53 expression is the primary predictor for the SUV_max_. Based on our findings, FDG-PET might be a simple and good non-invasive method for predicting p53-related chemotherapy resistance in NSCLCs. But cautions should be taken when using this method in the clinical settings.

## Competing interests

The authors declare that they have no competing interests.

## Authors’ contributions

XYD and YMG designed research; XYD, WW, JSW and SJ performed the research; XYD, WW and JGG analyzed data, XYD and WW wrote the paper. All authors read and approved the final manuscript.

## Pre-publication history

The pre-publication history for this paper can be accessed here:

http://www.biomedcentral.com/1471-2407/13/546/prepub

## Supplementary Material

Additional file 1: Figure S1 Multiple stepwise regression analysis of the primary predictor for SUV_max_. Click here for file
