# Supplementary figures and images for: Fluorodeoxyglucose positron emission tomography and chemotherapy-related tumor marker expression in non-small cell lung cancer
Source: BMC Cancer. 2013 Nov 15;13:546. doi: 10.1186/1471-2407-13-546 (PMC3835621; doi:10.1186/1471-2407-13-546)

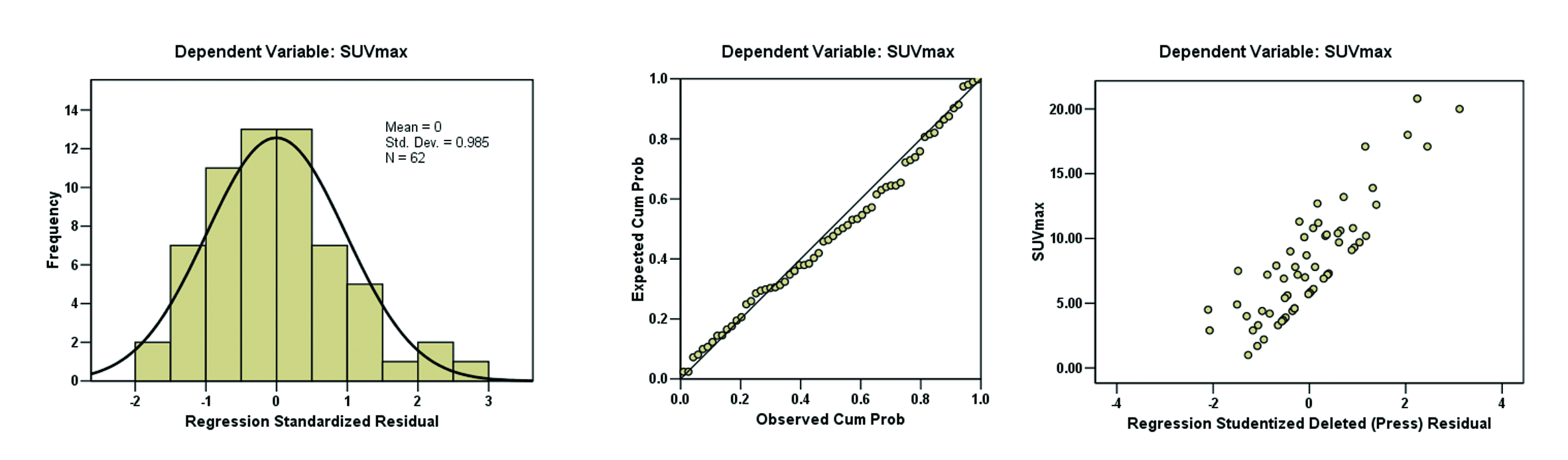

Supplement: Additional file 1: Figure S1 — Multiple stepwise regression analysis of the primary predictor for SUVmax. [file 1471-2407-13-546-S1.tif]
